# Extracellular Vesicles From Microalgae: Uptake Studies in Human Cells and *Caenorhabditis elegans*

**DOI:** 10.3389/fbioe.2022.830189

**Published:** 2022-03-24

**Authors:** Sabrina Picciotto, Pamela Santonicola, Angela Paterna, Estella Rao, Samuele Raccosta, Daniele Paolo Romancino, Rosina Noto, Nicolas Touzet, Mauro Manno, Elia Di Schiavi, Antonella Bongiovanni, Giorgia Adamo

**Affiliations:** ^1^ Cell-Tech HUB and Institute for Research and Biomedical Innovation, National Research Council of Italy (CNR), Palermo, Italy; ^2^ Department of Biological, Chemical and Pharmaceutical Sciences and Technologies, University of Palermo, Palermo, Italy; ^3^ Institute of Biosciences and BioResources (IBBR)—National Research Council of Italy (CNR), Naples, Italy; ^4^ Cell-Tech HUB and Institute of Biophysics, National Research Council of Italy (CNR), Palermo, Italy; ^5^ Centre for Environmental Research Innovation and Sustainability, Institute of Technology Sligo, Sligo, Ireland

**Keywords:** microalgae, extracellular vesicles, nanoalgosomes, *Caenorhabditis elegans*, cellular uptake

## Abstract

Extracellular vesicles (EVs) are lipid membrane nano-sized vesicles secreted by various cell types for intercellular communication, found in all kingdoms of life. Nanoalgosomes are a subtype of EVs derived from microalgae with a sustainable biotechnological potential. To explore the uptake, distribution and persistence of nanoalgosomes in cells and living organisms, we separated them from a culture of the chlorophyte *Tetraselmis chuii* cells by tangential flow filtration (TFF), labelled them with different lipophilic dyes and characterized their biophysical attributes. Then we studied the cellular uptake of labelled nanoalgosomes in human cells and in *C. elegans*, demonstrating that they enter the cells through an energy dependent mechanism and are localized in the cytoplasm of specific cells, where they persist for days. Our data confirm that nanoalgosomes are actively uptaken *in vitro* by human cells and *in vivo* by *C. elegans* cells, supporting their exploitation as potential nanocarriers of bioactive compounds for theranostic applications.

## Introduction

Adhering to principles and practices of environmental sustainability in nanotechnology manufacturing is a multifaceted issue with myriads of applications, which range from the development of natural nanomedical devices to novel green nanomaterials suitable for several industrial sectors. The production of environmentally sustainable nanomaterials requires responsible nano-manufacturing practices so as to minimise the use of toxic chemicals, to reduce waste and to generate less greenhouse gases (Nel et al., 2013). Efforts are ongoing to develop new nanomaterials to be utilised as nanocarriers for specific targets or controlled drug delivery for diagnosis and disease treatment, but also as ingredients for new cosmetic and nutraceutical formulations (Arrad et al., 1992; Adamo et al., 2016; Adamo et al., 2017).

There has been growing interest in microalgae, which are increasingly viewed as sustainable resources with applications in different fields (Sutherland et al., 2021). A range of microalgae species from varying lineages have been investigated for their potential to synthesise and accumulate value-added products with remarkable biological qualities (Orejuela-Escobar et al., 2021). For instance, it has been found that many microalgae can produce a variety of natural metabolites with high antioxidant potential (Tiwari et al., 2012; Zhu, 2015).

Within the VES4US consortium, we developed a platform for the production of extracellular vesicles (EVs), called nanoalgosomes, which are isolated from the cultivation medium of microalgal reactors (Adamo et al., 2021; Picciotto et al., 2021). EVs, which are lipid membrane nano-sized vesicles secreted by various cell types, play critical roles in inter-cellular as well as inter-species communication (Muraca et al., 2015; Soareset et al., 2017; Cai et al., 2018; Gill et al., 2019; Bleackley et al., 2020; Picciotto et al., 2020). Nanoalgosomes show several attributes expected from EVs in terms of morphology, size, distribution, protein content and immunoreactivity (Adamo et al., 2021; Picciotto et al., 2021). Moreover, nanoalgosomes offer a number of advantages compared to mammalian cell-, plant-, bacteria- or milk-derived EVs in that microalgal cells have high growth rates, can be cultured on non-arable land under controlled environmental conditions in photo-bioreactors, and can produce, in a renewable manner, nanoalgosomes with a yield comparable to those of other sources (Wang et al., 2013; Kim et al., 2015; Raimondo et al., 2015; Munagala et al., 2016; Bitto and Kaparakis-Liaskos, 2017; Gerritzen et al., 2017; Pocsfalvi et al., 2018; Paganini et al., 2019). In addition, the natural origin and sustainable trait of nanoalgosomes grant them greater societal acceptance and make them less subject to sensitive to ethical questions in the context of using them as new natural nano-materials.

Previous results have shown nanoalgosomes to be uptaken by different cellular systems and to be non-cytotoxic at the doses tested (Adamo et al., 2021; Picciotto et al., 2021). Here, a more detailed analysis is provided using different EV staining methods so as to better characterise their concentration, size distribution and cellular uptake *in vitro* (Verweij et al., 2021). Our knowledge is further extended by the use of an *in vivo* model organism *Caenorhabditis elegans* (Nematoda). This is carried out in the context of biosafety assessments in whole organisms of nanoparticles. As such, invertebrate *in vivo* assays have been recently considered highly suitable tests (Hunt, 2016; Wu et al., 2019). Unlike higher organisms, invertebrate models such as *C. elegans* are faster, less expensive and raise less ethical concerns for scientific research, hence fulfilling the 3R principles (Li et al., 2021). In addition, owing to its body transparency, *C. elegans* has been used to study nanoparticle uptake, toxicity and biodistribution (Scharf et al., 2013), to understand EVs secretion and function (Beer et al., 2016), or to explore the modulation of probiotic bacteria-derived EVs on host immune responses (Li et al., 2017).

Our data show that nanoalgosomes can be efficiently taken up by mammalian cells in culture and by *C. elegans* intestinal cells, suggesting a potential role in cross-kingdom communication.

## Materials and Methods

### Microalgae Cultivation

A stock culture of the marine chlorophyte *Tetraselmis chuii* CCAP 66/21b was grown in borosilicate glass flask in f/2 medium (Guillard, 1975) up to its exponential growth phase and then used, *via* a 10% v/v inoculum, to inoculate a 7.5 L culture in a photobioreactor PB 200 (GroTech GmbH, Germany) at an initial concentration of 0.5 mg/ml (wet weight). The cultures were maintained at a temperature of 20 ± 2°C, with a white light intensity of 100 μE m−2 s−1 and a 14:10 light/dark photoperiod for 30 days.

### Nanoalgosome Purification Methods: Tangential Flow Filtration

Nanoalgosome isolation was performed using the KrosFlo^®^ KR2i TFF System from Repligen (Spectrum Labs., Los Angeles, CA, United States) and three modified polyethersulfone hollow fiber membranes (S04-E65U-07-N, S04-P20-10-N and S04-E500-10-N, Spectrum Labs.). Briefly, after 30 days, the microalgae cultures were clarified by microfiltration using a 650 nm hollow fiber cartridge housed in the KrosFlo^®^ KR2i TFF System. Feed flow and transmembrane pressure (TMP) were kept constant at 450 ml/min and 0.05 bar, respectively. The first retentate (>650 nm sized particles) was concentrated into a final volume of 150 ml, and the 650 nm permeate (<650 nm sized particles) was then processed for a second microfiltration step using a 200 nm hollow fiber membrane at a 450 ml/min feed flow and 0.05 bar TMP. The resulting permeate (<200 nm sized particles) was processed for the last ultrafiltration step using a 500 kDa MWCO hollow fiber membrane with a feed flow of 450 ml/min and 0.05 bar TMP, prior to concentration to a final volume of 150 ml. Subsequently, the samples were further concentrated and diafiltrated seven times with PBS using a smaller 500-kDa cutoff TFF filter module (C02-E500-10-N, Spectrum Labs., MicroKros) and a feed flow of 75 ml/min and 0.25 bar TMP, returning a final volume of approximately 5 ml.

### Nanoalgosome Fluorescent Labelling

**FIGURE 1:**
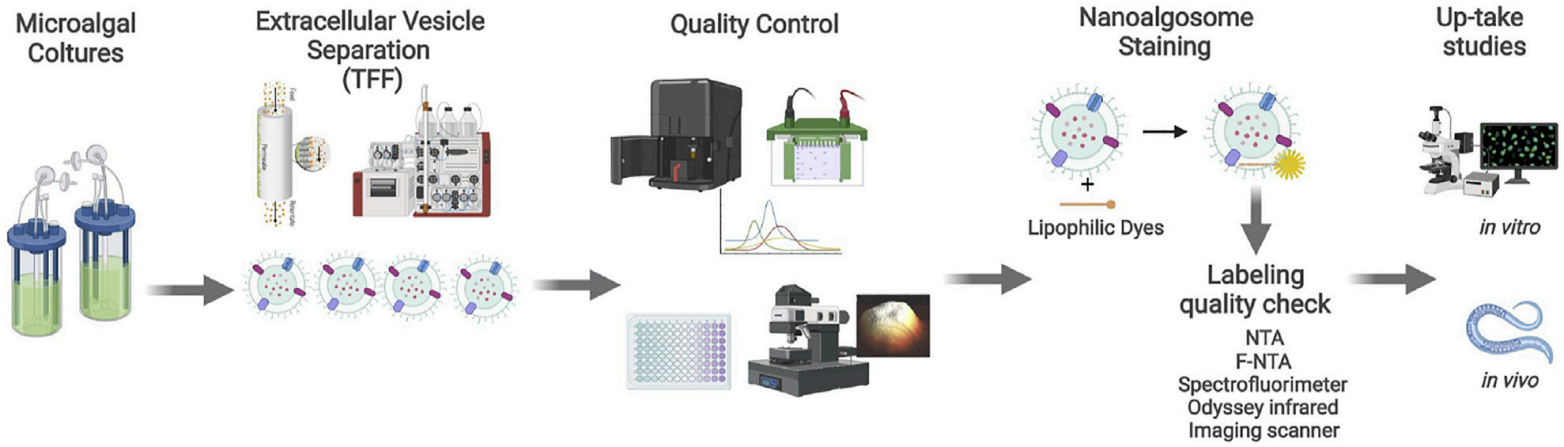
Nanoalgosome production workflow. Starting from the *T. chuii* culture medium, nanoalgosomes are separated by TFF and checked by quality control procedures. Downstream analyses include the staining of nanoalgosomes and *in vitro* and *in vivo* uptake studies*.* Created also with the support of BioRender.com.

The protein content of nanoalgosomes was measured using the micro-bicinchoninic BCA Protein Assay Kit (Thermo Fishers Scientific) (Romancino et al., 2018) and the nanoparticle size distribution and concentration were measured using a NanoSight NS300 (Malvern Panalytical, United Kingdom) as described in Adamo et al., 2021. After performing nanoalgosome quality control checks (Figure 1), EV labelling was carried out using three specific lipophilic dyes (Di-8-Anepps, PKH26 and DiR).

Nanoalgosome staining with the Di-8-Anepps was performed as in Adamo et al., 2021. Briefly, 500 nM of Di-8-Anepps (Invitrogen, filtered 20 nm with Anotop filter) was incubated with 5 × 10^10^ particles/mL for 30 min at room temperature. The samples were dialysed (Slide-A-Lyzer MINI Dialysis Device, 3.5 KDa MWCO, Thermo Fishers Scientific) against PBS to remove unbound fluorophore. The red fluorescent staining of nanoalgosomes was carried out using PKH26, a fluorescent cell linker used for cell membrane labelling (Ex/Em: 551/567 nm, Sigma-Aldrich). As for Di-8-Anepps, PKH26 fluorescence is activated in apolar environments and is specifically enhanced when bound to the lipid membrane of cells or EVs. The third lipophilic dye, DiR (1,1′-Dioctadecyl-3,3,3′,3′-Tetramethylindotricarbocyanine Iodide, Invitrogen, filtered 20nm, Anotop filter) is weakly fluorescent in H_2_O but fluorescent and photo-stable when incorporated into lipid membranes. Prior to staining, PKH26 in the Diluent C (supplied by Sigma-Aldrich with PKH26) vehicle was incubated at 37°C for 15 min to a final concentration of 3 μM (dye solution), while DiR was used at 0.5, 1, 3.5 μM (dye solutions). In parallel, particle-free PBS was used as a control for both dyes, using the specific amount of free probe for each. For the labelling of nanoalgosomes, 5 × 10^10^ particles/mL were incubated with dye solutions. After 1 h at room temperature, the stained nanoalgosomes were diluted to 3 ml with PBS and pelleted by ultracentrifugation at 118,000 × g for 70 min at 4°C using a Beckman SW55Ti rotor to remove the unbound dyes (Wiklander et al., 2015; Pužar et al., 2018). The pellet was gently resuspended in PBS overnight at 4°C. The quality check for Di-8-Anepps fluorescent nanoalgosomes was then monitored by Fluorescent Nanoparticle Tracking Analysis (NTA) using the Nanosight NS300 instrument (Malvern Panalytical, Malvern, United Kingdom). Dilution of the sample in PBS was performed in order to adjust the range of particles per frame to the working range of the system (10^8^ particle/mL). A total of five videos were captured at a syringe speed of 60 in light scattering and 150 in fluorescence modes. Data were further processed using the NanoSight Software NTA 3.1 Build 3.1.46 with a detection threshold of 5. The absence of significant fluorescent signal after dialysis on labelled control samples, at an equivalent fluorophore concentration used to that of the labelled nanoalgosomes, was confirmed (data not shown). Quality check for PKH26-labelled nanoalgosomes was verified by spectrofluorimetric analysis (Spectrofluorometer Jasco fp-6500) and the size distribution and concentration were checked with NTA. The staining efficiency of DiR-labelled nanoalgosomes was verified by Odyssey infrared Imaging System (LiCor Biosciences, software V 3.0) and the size distribution and concentration were checked with NTA.

### *In vitro* Cellular Uptake of Nanoalgosomes

#### Cell Cultures

MDA-MB 231, an epithelial, human breast cancer cell line, was maintained at 37°C in a humidified atmosphere (5% CO_2_) in Dulbecco’s Modified Eagle Medium (DMEM) (Sigma-Aldrich) containing 10% (v/v) Fetal Bovine Serum (FBS) (Gibco, Life Technologies) plus 2 mM L-glutamine, 100 U/mL penicillin and 100 mg/ml streptomycin (Sigma-Aldrich).

#### Cellular Uptake Study

For the PKH26-labelled nanoalgosome uptake experiment, the MDA-MB 231 cell line was grown at a density of 5 × 10^4^ cells/well in 12-well plates containing sterile coverslips in complete medium for 24 h. Cells were then incubated with different amounts of nanoalgosomes (10 and 20 μg/ml) at 37°C or 4°C, as well as with an equivalent amount of the control samples. After different incubation times (3, 6 and 24 h), cells were washed twice with PBS, fixed with 3.7% paraformaldehyde for 15 min and washed again twice with PBS. The nuclei were then stained with VECTASHIELD Mounting Medium with DAPI.

For the DiR-labelled nanoalgosome uptake experiment, MDA-MB 231 cells were plated at a density of 4 × 10^3^ cells/well in 96-well plates in complete medium for 24 h. Cells were then incubated with different amount of nanoalgosomes (2 and 10 μg/ml, respectively stained with of 0.5, 1, 3.5 μM of DiR) at 37°C or 4°C, as well as with an equivalent amount of control samples. After different incubation times (3, 6 and 24 h), the fluorescence intensities inside the cells were monitored in real time, after removing the culture medium, with the Odyssey infrared Imaging System (LiCor Biosciences, software V 3.0). Cell viability assay was performed as previously described in Adamo et al*.,* 2021, incubating MDA MB 231 cells with 10 and 20 μg/ml of nanoalgosomes for 24 and 48 h. All experiments were performed in three independent biological replicates. Statistical analysis was performed using a One-way ANOVA calculated by Statistics Kingdom online software.

#### Fluorescence and Confocal Microscopy Analysis

The PKH26-labelled nanoalgosomes cellular localization was monitored by fluorescence microscopy analysis (Nikon Eclipse 80i) and confocal laser scanning microscopy (Olympus FV10i; a 1 μm thick optical section was taken from a total of about 15 sections for each sample). The orthogonal projection of the Z-stack was obtained using the imageJ software.

### *In vivo* Cellular Uptake of Nanoalgosomes

#### Animal Culture

Nanoalgosome uptake was assessed in wild type *C. elegans* strain N2, Bristol variety and in HA2031 strain, harboring *rtIs31* transgene that expresses GFP in the intestinal nuclei. These strains were provided by the *Caenorhabditis* Genetics Center (CGC), which is funded by the NIH Office of Research Infrastructure Programs (P40 OD010440). Animals were grown and handled following standard procedures under uncrowded conditions on nematode growth medium (NGM) agar plates seeded with *Escherichia coli* strain OP50, unless differently specified (Brenner, 1974).

#### *In vivo* Uptake of Nanoalgosomes by *C. elegans*

For the soaking experiments, thirty synchronized animals, at L4 larval stage, were manually transferred into a medium composed of OP50 bacteria, M9 buffer, antibiotic antimycotic solution (2x) (cat. n. A5955 Sigma-Aldrich^®^) and cholesterol (5 ng/ml), supplemented with Di-8-Anepps-nanoalgosomes (12 μg/ml or 20 μg/ml final concentrations) in a 96 multi-well plate (70 µl final volume/well). After 3, 6 and 24 h of treatment in the dark and mild agitation on a swinging oscillator (15 rpm), the animals were transferred to clean NGM plates with bacteria to allow the animals to crawl for 1 h, so as to remove the excess of dye.

For the *in solido* experiments, twenty synchronized L4 larvae were transferred on freshly prepared NGM plates seeded with heat-killed OP50 bacteria and Di-8-Anepps-nanoalgosomes (20 μg/ml, final concentration), PKH26-nanoalgosomes (45 μg/ml), Di-8-Anepps (2.5 µM), PKH26 (7.5 µM), PBS buffer or unstained nanoalgosomes (20 μg/ml). The dilutions were performed considering the final volume of NGM (4 ml), meaning that to obtain a final concentration of 20 μg/ml of nanoalgosomes, 80 µg of EVs corresponding to ∼4 × 10^12^ total particles were added to the solified agar in the plates. After 24 h of exposure in the dark, young adult animals were transferred to clean NGM plates with OP50 bacteria to let the animals crawl for 1 h and remove the excess of dye.

For the injection experiments, ten animals were injected and were analyzed after 24 h. Young-adult animals were immobilised on agar pads with halocarbon oil 700 (cat. n.H8898 Sigma-Aldrich^®^) and injected in the pseudocoelom cavity near the pharynx (avoiding the intestine) with Di-8-Anepps-nanoalgosomes (20 μg/ml) (Mello et al., 1995; Mohan et al., 2010). Animals were then recovered on clean NGM plates with OP50 bacteria for 24 h. All experiments were performed in triplicates using at least two independent nanoalgosomes preparations.

#### Fluorescence and Confocal Microscopy Analysis

After allowing the animals to eliminate the excess of dye, they were transferred on glass slides with 4% agar pads and immobilized alive for microscopy analysis with 0.01% tetramisole hydrochloride (cat. n. T1512 Sigma-Aldrich^®^). Confocal and epi-fluorescence (FITC filter) images were collected with Leica TCS SP8 AOBS microscope using a 40x objective. For persistence analysis, the animals were collected and observed after 24, 48 and 72 h from the treatment.

## Results

### Setting up of Efficient Staining of Nanoalgosomes Using Three Different Dyes

**FIGURE 2:**
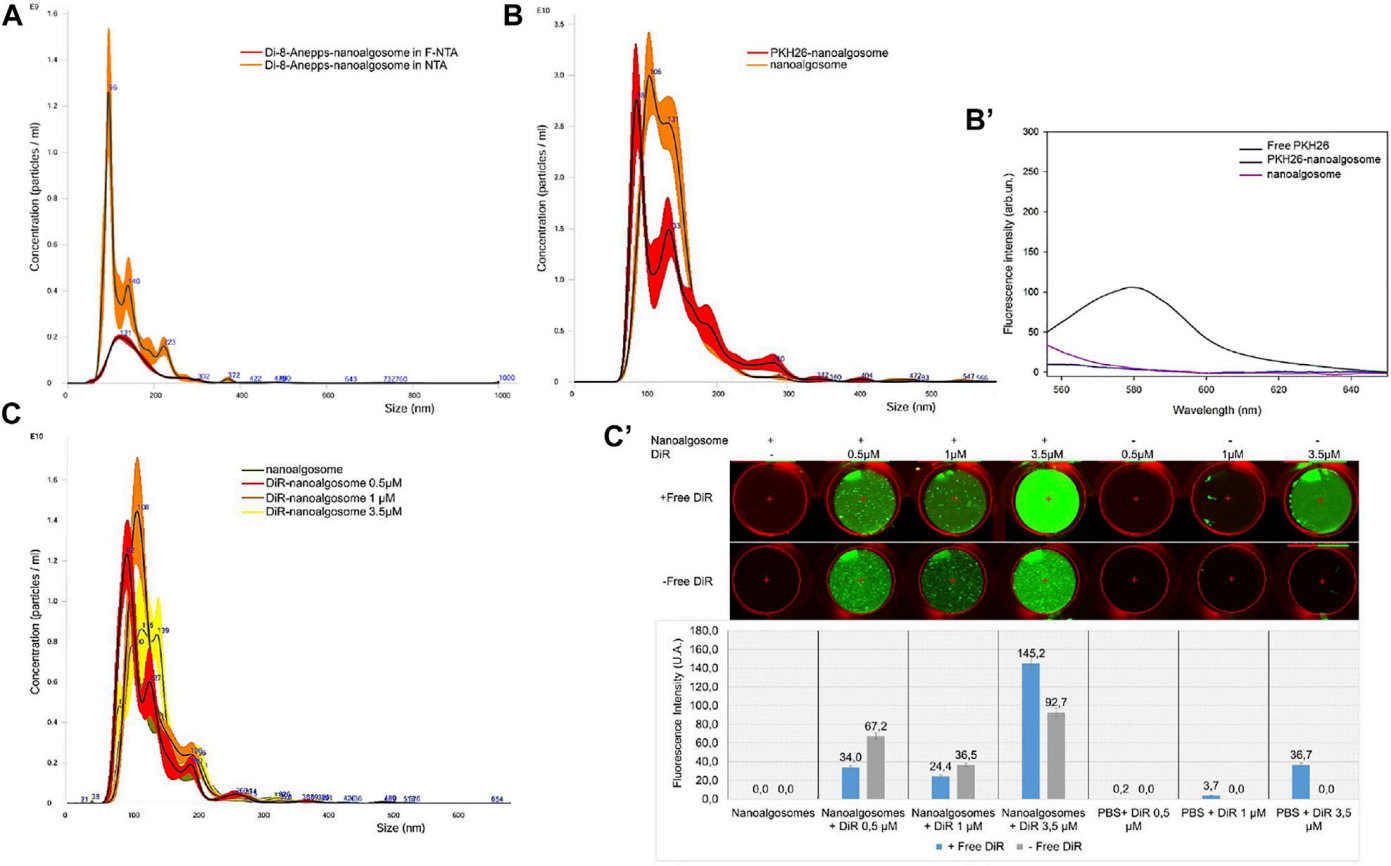
Fluorescent staining of nanoalgosomes with three different dyes **(A)** Fluorescent-NTA (F-NTA) and standard (scattering mode) NTA to determine the size distribution and concentration of nanoalgosomes stained with Di-8-ANEPPS. This comparative analysis gives the concentration of fluorescent nanoparticles, excluding non-vesicle contaminants. **(B)** Size distributions and concentrations by NTA of PKH26-labelled nanoalgosomes and unstained nanoalgosomes, showing a largely overlapping distribution. **(B’)** Fluorescent emission spectra of PKH26-labelled nanoalgosomes compared to PKH26 free dye and unstained nanoalgosomes. **(C)** Size distribution and concentration analyses by NTA of nanoalgosomes stained with DiR at different concentrations show no variation compared to unstained nanoalgosomes after the free dye removal. **(C’)** Infrared fluorescent emission imaging and intensities (λ_800nm_) obtained using an Odyssey IR scanner of DiR-labelled nanoalgosomes and free dye at different concentrations before and after free dye removal.

Nanoalgosomes were isolated from the conditioned medium of a *T. chuii* culture by TFF and were characterised using biophysical and biochemical approaches (see Figure 1). As previously described, the nanoalgosomes were stained with Di-8-Anepps, a lipophilic dye which is non-fluorescent until bound to membranes, to verify the presence of lipid bilayer-nanovesicles, and in turn to check the quality of nanoalgosome preparations (Adamo et al., 2021). Here, we assessed the uptake of Di-8-Anepps-labelled nanoalgosomes in *C. elegans*. Further, two additional nanoalgosome staining strategies were considered using red and infrared lipophilic fluorescent dyes for future applications in potency assays *in vitro* and *in vivo* (Gangadaran et al., 2018)*.* A quality check of the staining procedure was performed by NTA and no variations in size and concentration were obtained for the nanoalgosomes labelled with the three lipophilic dyes compared to the unstained nanoalgosome controls (Figure 2).

We monitored the size distribution of the fluorescent Di-8-Anepps-nanoalgosomes cleaned from the free dye using NTA in fluorescence and light scattering (Figure 2A). In this way, we excluded artifacts, like protein aggregates, nanobubbles and insoluble salts, which returned a green fluorescent nanoalgosome population (54% of the total nanoparticles as measured by light scattering) with the same size distribution and mode of the unstained control.

NTA operated in standard scattering mode was also used for the PKH26- and DiR-based nanoalgosome staining to compare concentrations and size distributions between the original and stained samples. After PKH26-labelling, the nanoalgosome size distribution and concentration remained constant at 88 ± 2.5 nm and 1.8 × 10^12^ ± 9.8 × 10^10^ particles/mL, respectively, confirming the absence of aggregates (Figure 2B). Figure 2B’ shows the comparison of the fluorescence emission spectra of PKH26-labelled nanoalgosomes, unstained nanoalgosomes and free dye. Neither the raw nanoalgosomes nor the free dye emitted fluorescence when excited (λ_551nm_) whereas PKH26-labelled nanoalgosomes showed high red fluorescent emission (λ_567nm_), confirming successful staining.

The DiR dye was selected based on its high stability in biological fluid for future *in vivo* applications. Based on literature data reported for the DiR staining of mammalian cell-derived extracellular vesicle (Gerwing et al., 2020; Samuel et al., 2021; Verweij et al., 2021), we tested three different starting concentrations of the DiR dye (0.5, 1, 3.5 µM) to optimise the labelling of nanoalgosomes. The size distribution and concentration of the nanoalgosomes did not undergo changes following the removal of the free dye compared to unstained nanoalgosomes (Figure 2C). Figure 2C’ shows the fluorescence emission images (λ_800nm_) obtained using an Odyssey infrared Imaging scanner, before and after removal of the free dye. A more effective staining of nanoalgosomes was obtained using 3.5 µM of DiR, which returned a higher fluorescence intensity compared to the other two other concentrations (i.e., 0.5 and 1 μM) and the negative controls (i.e., free DiR that showed no detectable fluorescence) after free dye removal.

### *In vitro* Uptake of PKH26 and DiR Labelled Nanoalgosomes

**FIGURE 3:**
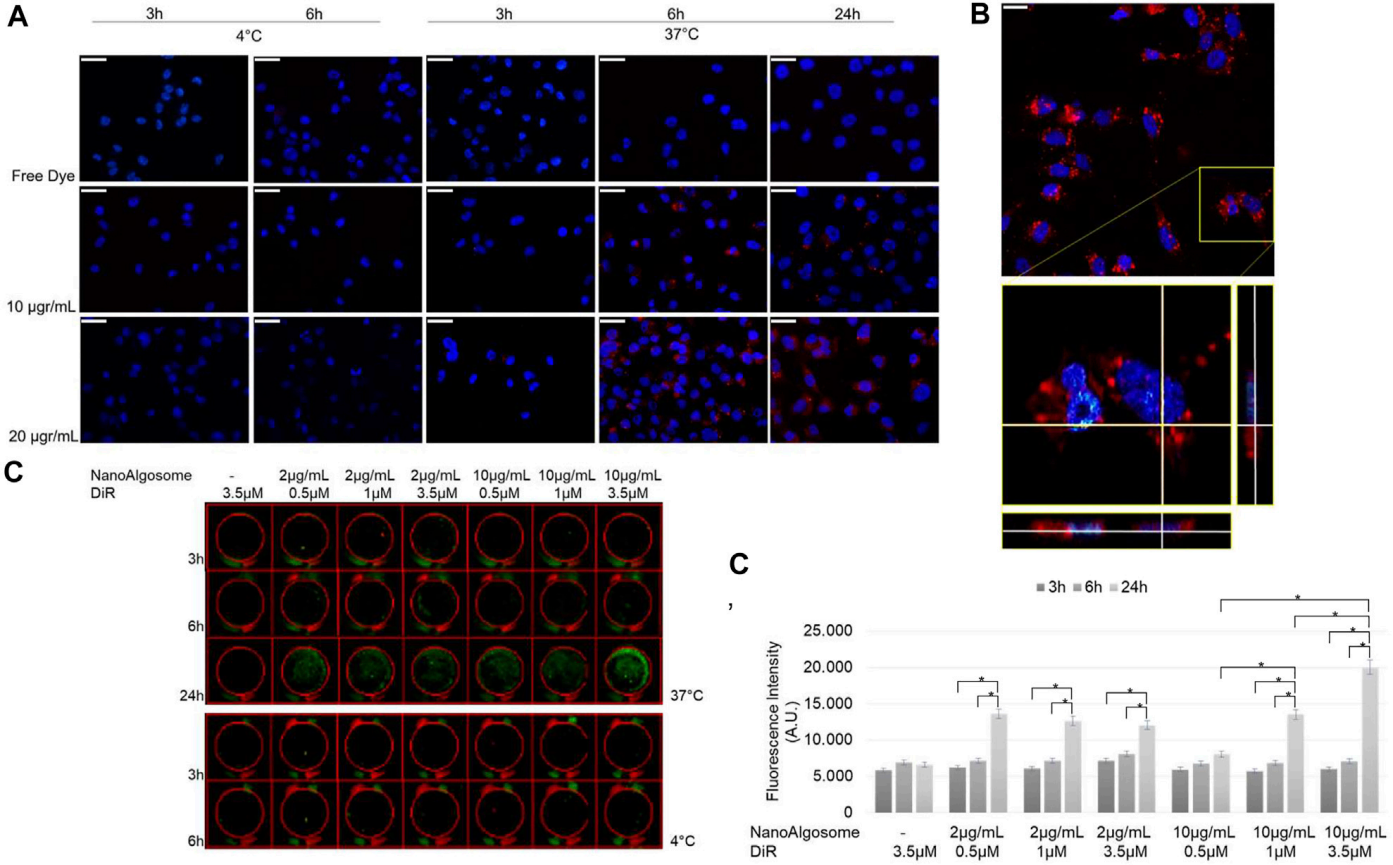
Nanoalgosome cellular uptake *in vitro*.**(A)** Representative fluorescence microscopy images showing the cellular uptake of PKH26-fluorescent nanoalgosomes (red) in MDA-MB 231 cells (nuclei in blue) incubated with different concentrations of PKH26-labelled nanoalgosomes (10 and 20 μg/ml) at 37°C for 3, 6 and 24 h. The free dye control and 4°C incubations are shown as negative controls (Magnification 40X). Scale bar 50 µm. **(B)** Confocal microscopy analysis of PKH26-labelled nanoalgosome internalisation in MDA-MB 231 cells (nuclei in blue) incubated with 20 μg/ml of red fluorescent nanoalgosomes at 37°C for 24 h. The inset of confocal Z-stack acquisition shows its orthogonal projections at a focal depth of 9 μm over a total scanning thickness of ∼18 μm (Magnification 60X). Scale bar 25 µm. **(C)** Representative infra-red scanner images showing the cellular uptake of DiR-labelled nanoalgosomes (green) in MDA-MB 231 cells incubated with different concentrations of DiR-labelled nanoalgosomes at 37°C for 3, 6 and 24 h. The corresponding IR fluorescence intensities are measured in triplicate (*p < 0.0001) and reported **(C’)** through the in-cell function of Odyssey V3.0 software. Free dye and 4°C incubations are shown as negative controls.

To study the intracellular uptake of isolated *T. chuii*-derived nanoalgosomes, we used PKH26 and DiR fluorescent dyes that were incorporated into the lipid membrane of nanoalgosomes. We previously demonstrated the biocompatibility of nanoalgosomes as well as the cellular uptake using nanoalgosomes stained with Di-8-Anepps, demonstrating that they are internalised by human cells over time through an energy dose-dependent mechanism (Adamo et al., 2021). First, we studied the uptake of red-fluorescent nanoalgosomes using MDA MB 231 cells treated with different doses (10–20 μg/ml), for 3, 6 and 24 h at 37 and 4°C (Figure 3). Moreover, we verified that incubation with nanoalgosomes (10 and 20 μg/ml up to 48 h) did not affect cell viability or induce cell proliferation in MDA MB 231 cells (Supplementary Figure S1). In Figure 3A, epifluorescence images resolve the positions of PKH26 labelled nanoalgosomes within tumor cells. Dose- and time-dependent uptakes were observed. The uptake increased with incubation time at 37°C, while it was inhibited by incubation at 4°C, indicating an energy-dependent endocytic process. The images in Figure 3A show a low PKH26-nanoalgosome internalisation for short incubation times (i.e., 3 h). After 6 h of incubation, the amount of red fluorescent nanoalgosomes within the cells increased, reaching a maximum cytoplasmic/intracellular concentration after 24 h. No red fluorescent signal was observed for MDA-MB 231 cells incubated with the controls (staining-control samples after free dye removal) for all duration and temperature treatments used.

Confocal microscopy analyses supported the specificity of PKH26-labelled nanoalgosome uptake as the orthogonal projections showed evident intracellular localisation, in the mid-section focal plan of the cytoplasm closed to the nucleus (Figure 3B).

Finally, to assess the DiR-labelled nanoalgosome uptake *in vitro*, the cellular internalisation was compared across the doses and durations tested. The Odyssey images showed that DiR-labelled nanoalgosomes were internalised over time, in a dose- and energy-dependent manner, as observed with the other two dyes (Figure 3C).

The fluorescence intensities measured in real time inside the tumor cell line for all tested conditions at 37°C are shown in Figure 3C’. Interestingly, there was a higher level of internalisation for the cells incubated with 10 μg/ml of nanoalgosomes stained with 3.5 μM of DiR, after 24 h of incubation compared to the other tested conditions.

With these experiments we set-up a protocol for the best staining strategy of nanoalgosomes using three different fluorescence dyes, we confirmed the cellular uptake of nanoalgosomes and we established the best conditions for further *in vivo* experiments. Specifically, near-infrared dye is suitable for non-invasive *in vivo* applications because of their high signal-to-noise ratio, low autofluorescence of biological tissue in the 700–900 nm spectral range, and deep tissue penetration of the near-infrared light.

### *In vivo* Cellular Uptake in *C. elegans*

**FIGURE 4:**
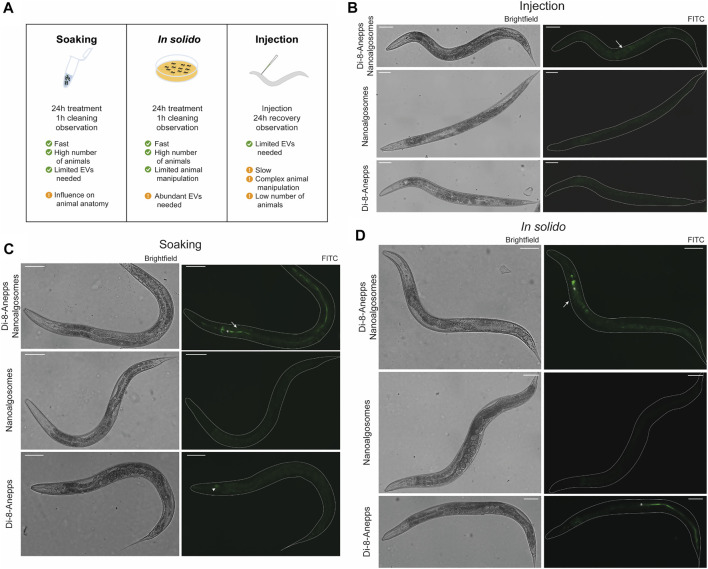
Set-up of labelled nanoalgosomes treatment in *C. elegans.* **(A)** Schematic representation of the administration methods used for testing the uptake in *C. elegans* of fluorescent nanoalgosomes. The best conditions used for treating animals as well as some advantages and disadvantages are listed. **(B–D)** Brightfield (left) and epifluorescence (with FITC filter, right) images of animals treated with Di-8-Anepps-nanoalgosomes, nanoalgosomes and free-dye (Di-8-Anepps) by injection **(B)**, soaking **(C)** and *in solido* **(D)**. A fluorescent signal was observed in the intestinal cells of the animals treated with labeled nanoalgosomes (arrows). Moreover, aspecific signals were observed in the head after soaking (arrowhead) and *in solido* with free-dye (asterisks). Anterior is left and ventral is down. Scale bar 75 µm.

**FIGURE 5:**
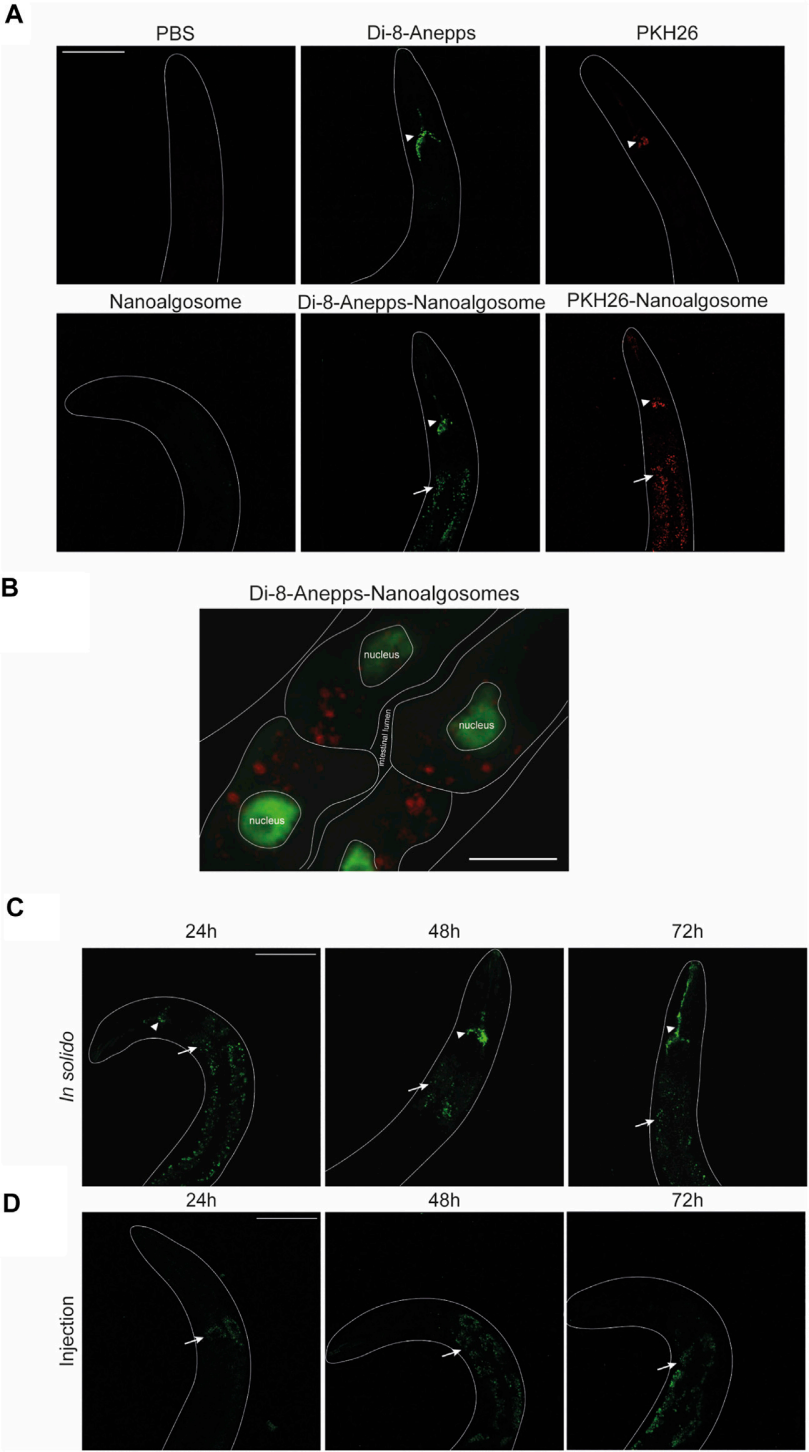
*C elegans* intestinal cells uptake and persistence of labelled nanoalgosomes.**(A)** Confocal images of animals treated with PBS, Di-8-Anepps free dye, PKH26 free dye for 24 h are shown in the upper panels; animals treated for 24 h with unstained nanoalgosomes, Di-8-Anepps-nanoalgosomes and PKH26-nanoalgosomes are shown in the lower panels. A fluorescent signal was observed in animals treated with labelled nanoalgosomes in the intestinal cells (arrows) and in the head (arrowheads). Anterior is up. Scale bar 75 µm. **(B)** Localisation of Di-8-Anepps-nanoalgosomes in the cytoplasm of intestinal cells expressing GFP in the nuclei thanks to *elt-2* promoter. The Di-8-Anepps-nanoalgosomes specific signal has been pseudo-colored in red. Scale bar 25 µm. **(C,D)** The persistence of the fluorescent signal (arrows) was assessed in animals treated with Di-8-Anepps-nanoalgosomes for 24, 48 and 72 h after treatments *in solido* **(C)** or injection **(D)**. A fluorescent signal in the head (arrowheads) is also visible in solido. Anterior is up. Scale bar 75 µm.

The animal model *C. elegans* was used for testing exogenous EVs uptake, distribution and persistence. First, we tested different times of treatment (3, 6 and 24 h) and two concentrations (12 μg/ml and 20 μg/ml) of Di-8-Anneps-labelled nanoalgosomes. Green fluorescent signal was observed only in the intestine of the animals (Figure 4). The best condition in terms of brightness was obtained using 20 μg/ml dose for 24 h treatment. In this case, the labelled particles were administered by immersing the animals in the nanoalgosome-containing solution (soaking), which allowed to rapidly test a high number of animals using several conditions at once together with limited manipulation and less nanoalgosomes being needed (Figures 4A,C). Then, to identify the best conditions to study EV uptake in a whole living animal, we compared three different administration methods: soaking (*in liquido*)*, in solido*, and injection. These methods offer advantages and disadvantages in terms of costs, time, physiology of the animals, quantity and concentration of EVs, and number of animals treated (Figure 4A). After treating the animals with Di-8-Anneps-labelled nanoalgosomes a fluorescent signal was observed in their intestine in all conditions (Figures 4B–D). A faint fluorescent signal was also observed in the head of the animals when using the *in solido* administration (Figure 4D). To confirm the specificity of the fluorescence observed, Di-8-Anepps free dye and unlabeled nanoalgosomes were used as controls for all the administration methods and no signal was visible, except in the lumen *in solido* and faintly in the head after soaking (Figures 4B–D). The treatments did not affect animal survival with all concentrations and methods tested. *In solido* and injection administrations showed similar labelling in the animals analysed, while soaking was less efficient and not all the animals were labelled; moreover a fluorescent signal was also observed in the intestinal lumen. Since injection is a time-consuming technique, allowing the observation of only few animals, the *in solido* method was used for further analyses. The whole animal body was observed at higher magnification, but the fluorescent signal appeared mostly confined to intestinal cells (Figure 5A). Confocal images confirmed the observations, highlighting a punctuate fluorescent signal in the intestinal cells and in the head of all animals treated with Di-8-Anepps-nanoalgosomes, but no signal was observed in the animals treated with PBS or unlabelled nanoalgosomes (Figure 5A). An unexpected fluorescent signal was instead observed in the head of the animal, but not in the intestinal cells, after treatment with Di-8-Anepps free dye, albeit using higher concentrations compared to the dye incorporated in nanoalgosomes (arrowheads in Figure 5A). To further confirm our observations, animals were treated with nanoalgosomes stained with the lipophilic PKH26 dye for 24 h and similar results were observed (Figure 5A). *C. elegans* body transparency allows the visualization of fluorescent proteins in specific tissues or cells when expressed through transgenics in living whole animals. Thus, we performed confocal analysis, on transgenic animals expressing the GFP only in intestinal nuclei after treatment with Di-8-Anepps-nanoalgosomes, confirming that the fluorescent signal is localized only in the cytoplasm of the intestinal cells (Figure 5B). The persistence of the fluorescent signal in a living whole organism was assessed by observing animals at 24, 48 and 72 h after injection or after interrupting the treatment *in solido*. Using both approaches, we observed that the specific intestinal fluorescent signal did persist, albeit becoming fainter after 72 h post-treatment; interestingly this time-window coincides with the entire fertile period of adult animals (Figures 5C,D).

We demonstrated that *C. elegans* intestinal cells uptake labelled extracellular vesicles and this fluorescence persists for 3 days; thus nanoalgosomes can be recognized and internalised in living organisms.

## Discussion

In this study, we explored the labelling of nanoalgosomes using three lipophilic dyes with different fluorescence emissions (green, red and infrared) and the monitoring of their uptake with *in vitro* and *in vivo* assays. The use of lipophilic dyes to study EV biogenesis and to carry out functional studies is quite common (Verweij et al., 2021). Microalgal derived extracellular vesicles have recently been characterised (Picciotto et al., 2021). Here, we report the isolation of nanoalgosomes from *T. chuii* culture medium by TFF, which were successfully labelled with three lipophilic dyes. The stained nanoparticles were then characterised in terms of size, concentration and fluorescence intensity using NTA, spectrofluorometric and infrared analyses. PKH26 and DiR labelled nanoalgosomes were successfully internalised by cultured tumoral cells in a way similar to that recently described for Di-8-Anepps labelled nanoalgosomes (Adamo et al., 2021). Epi-fluorescence microscopy confirmed that MDA MB231 cells can internalise PKH26-positive nanoalgosomes in a dose-, time- and energy-dependent manner. Subsequent confocal microscopy analysis of tumor cells revealed numerous red fluorescent puncta in the cell cytoplasm, while the orthogonal views showed the intracellular localisation of labeled nanoalgosomes closed to nucleus. The same uptake pattern was observed for nanoalgosomes labelled with the DiR dye.

The biological model system *C. elegans* was used to ascertain, in a living multicellular-organism, whether unstained nanoalgosomes can be uptaken and in which body parts they accumulate*.* Since we evaluated for the first time, to the best of our knowledge, the uptake of exogenous labelled EVs in *C. elegans*, we initially compared three administration routes previously used for the delivery of nanoparticles (Mohan et al., 2010; Zhang et al., 2011; Perni et al., 2017). We successfully observed for the three approaches tested a specific fluorescent signal in the cytoplasm of intestinal cells, with minor differences among the three administration methods. By using these different administration methods we demonstrated that EVs could be uptaken by both apical and basolateral membrane of the intestinal cells, in line with the high endocytic and exocytic trafficking capability of the intestinal cells (Sato et al., 2014). Following the exposure of the animals to labelled nanoalgosomes *via* the *in solido* method, we also observed a fluorescent signal in the head, possibly in the neurons, which are mainly concentrated at the anterior end of the animals and able to sense the environment through *cilia*. A similar observation was made using a very high concentration of free dyes, which can be explained by the fact that lipophilic dyes have been extensively used in *C. elegans* to label amphid neurons (Starich et al., 1995). Thus we cannot exclude that the labelling of neurons can be specifically obtained with labelled nanoalgosomes. Nevertheless, using all the three methods we did not observe any fluorescent signal in other animal tissues, suggesting a tissue-specific uptake of the nanoalgosomes.

While the soaking and *in solido* exposure routes allow the simultaneous treatment and analysis of hundreds of animals, injection is performed one animal at a time, which it is time consuming and cannot be applied to many animals. On the other hand, considering the amount of starting material needed, both soaking and injection can be preferred as they require a lower amount of EVs compared to feeding. However, worms usually grow *in solido* and *in liquido* cultivation can alter animal anatomy and its physiology (Çelen et al., 2018). Considering the availability of nanoalgosomes and the importance of observing several animals under suitable physiological conditions, the *in solido* method was chosen as the preferred option. The specificity of the observed signal was assessed using controls and the two lipophilic free dyes Di-8-Anepps and PKH26, thus further confirming the robustness of our nanoalgosome labelling approach. Finally we demonstrated that *C. elegans*, with its body transparency and potent genetics, is a powerful model system to study exogenous EVs uptake, persistence and biodistribution.

The data gathered in the present study showed that three different methods can be used to stain efficiently nanoalgosomes for in *in vivo* and *in vitro* uptake studies, and further applied in functional studies as nano-delivery system.

## Data Availability

The raw data supporting the conclusion of this article will be made available by the authors, without undue reservation.

## Supplementary Material

Supplementary Figure S1 Cell viability assay of MDA MB 231 cells treated with nanoalgosomes (10 and 20 µg/mL) for 24 and 48 h.

Supplementary Figure S2 Additional pictures of animals treated with Di-8-Anepps-nanoalgosomes by soaking, *in solido* or injection. Intestinal cells have uptaken the labelled EVs (arrows), and some are still visible in the lumen after soaking (asterisk). Brightfield (left) and epifluorescence with FITC filter images are shown (right). Upper panel shows a young adult animal; in the middle panel an L4 stage larva is depicted; the lower panel shows an old adult with several eggs in the uterus. Scale bar 75 µm. Anterior is left, ventral is down.
