# Supplementary figures and images for: Extracellular Vesicles From Microalgae: Uptake Studies in Human Cells and Caenorhabditis elegans
Source: Front Bioeng Biotechnol. 2022 Mar 24;10:830189. doi: 10.3389/fbioe.2022.830189 (PMC8987914; doi:10.3389/fbioe.2022.830189)

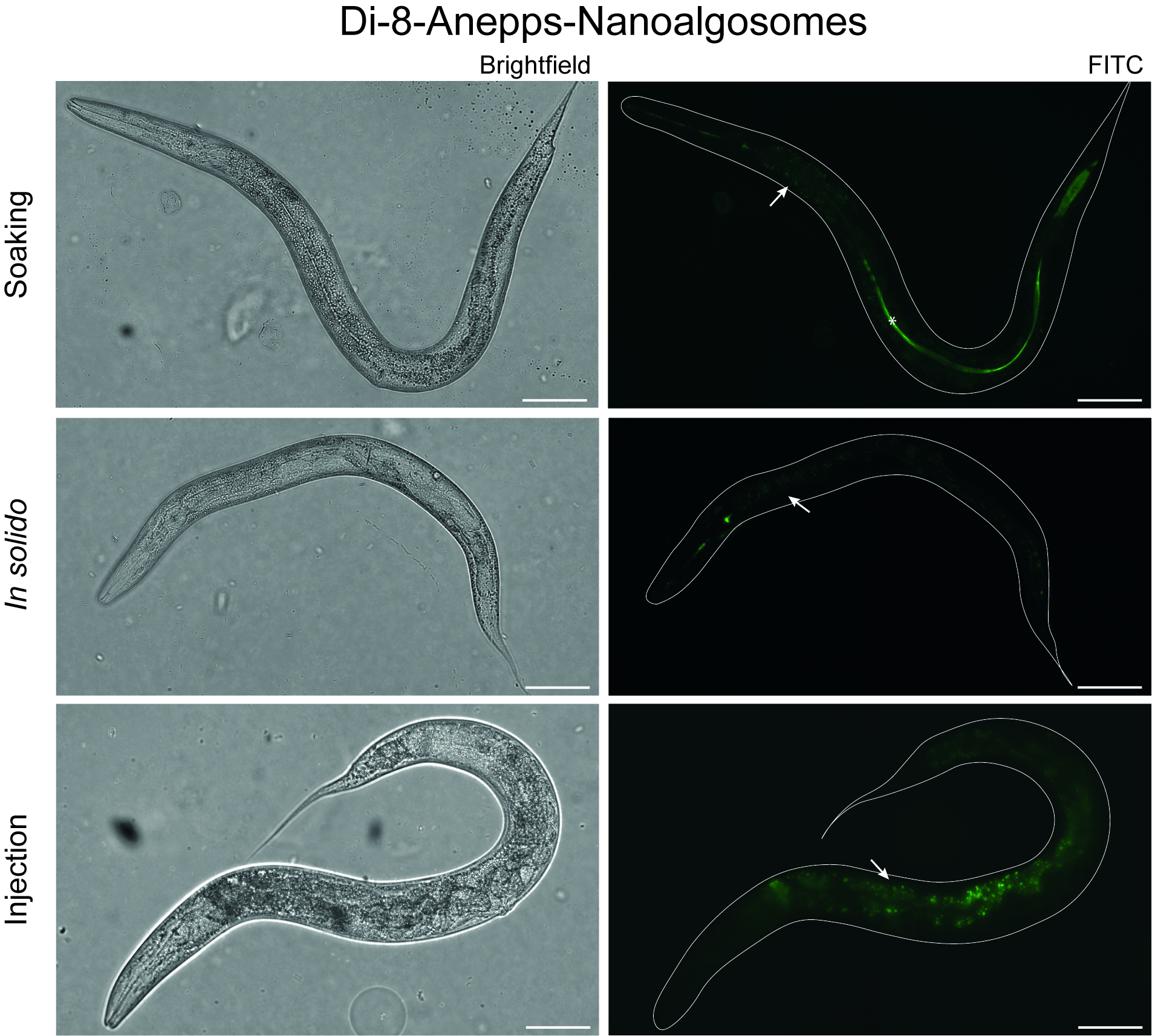

Supplement: Supplementary file 1 [file Image2.TIF]

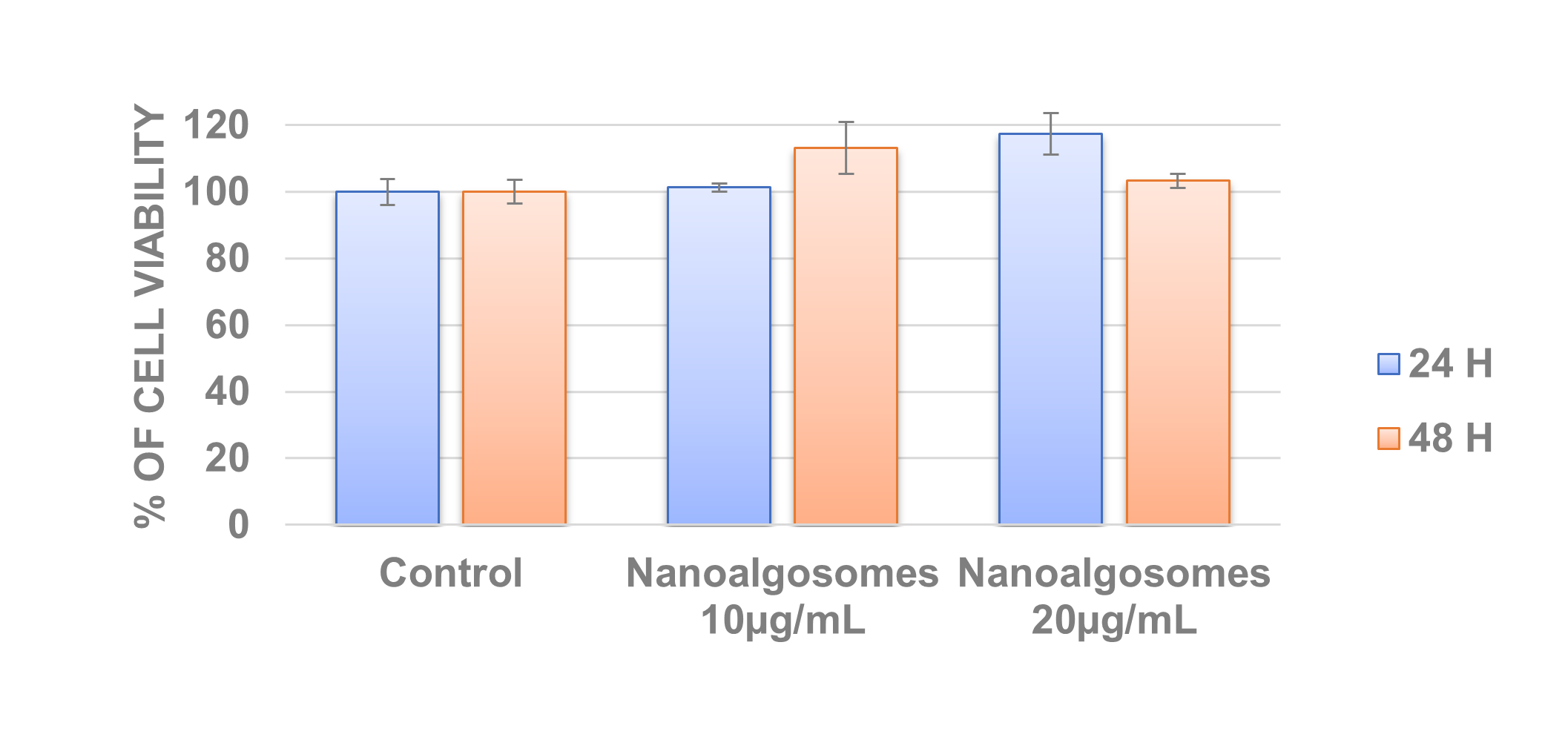

Supplement: Supplementary file 2 [file Image1.TIF]
